# A truncating *PET100* variant causing fatal infantile lactic acidosis and isolated cytochrome *c* oxidase deficiency

**DOI:** 10.1038/ejhg.2014.214

**Published:** 2014-10-08

**Authors:** Monika Oláhová, Tobias B Haack, Charlotte L Alston, Jessica AC Houghton, Langping He, Andrew AM Morris, Garry K Brown, Robert McFarland, Zofia MA Chrzanowska-Lightowlers, Robert N Lightowlers, Holger Prokisch, Robert W Taylor

**Affiliations:** 1 Wellcome Trust Centre for Mitochondrial Research, Newcastle University, Newcastle upon Tyne, UK; 2 Institute of Human Genetics, Helmholtz Zentrum München, Neuherberg, Germany; 3 Institute of Human Genetics, Technische Universität München, Munich, Germany; 4 Willink Biochemical Genetics Unit, Manchester Centre for Genomic Medicine, Central Manchester University Hospitals NHS Foundation Trust, Manchester, UK; 5 Department of Biochemistry, University of Oxford, Oxford, UK

## Abstract

Isolated mitochondrial complex IV (cytochrome *c* oxidase) deficiency is an important cause of mitochondrial disease in children and adults. It is genetically heterogeneous, given that both mtDNA-encoded and nuclear-encoded gene products contribute to structural components and assembly factors. Pathogenic variants within these proteins are associated with clinical variability ranging from isolated organ involvement to multisystem disease presentations. Defects in more than 10 complex IV assembly factors have been described including a recent Lebanese founder mutation in *PET100* in patients presenting with Leigh syndrome. We report the clinical and molecular investigation of a patient with a fatal, neonatal-onset isolated complex IV deficiency associated with multiorgan involvement born to consanguineous, first-cousin British Asian parents. Exome sequencing revealed a homozygous truncating variant (c.142C>T, p.(Gln48*)) in the *PET100* gene that results in a complete loss of enzyme activity and assembly of the holocomplex. Our report confirms *PET100* mutation as an important cause of isolated complex IV deficiency outside of the Lebanese population, extending the phenotypic spectrum associated with abnormalities within this gene.

## Introduction

Mitochondrial oxidative phosphorylation (OXPHOS) is the primary pathway for adenosine triphosphate (ATP) production in eukaryotic cells. This OXPHOS system comprises five transmembrane complexes (I–V) consisting of ~90 protein subunits that are encoded by either the mitochondria's own genetic material (mtDNA) or the nuclear genome. Of these, complexes I–IV constitute the respiratory chain and complex V, the ATP synthase. Mitochondrial respiratory chain disease is caused by defective OXPHOS and represents a major inborn error of metabolism.^1^ Mitochondrial disease is associated with both a varied age of onset and a diverse spectrum of clinical presentations in which brain, CNS and muscle involvement are common.^2^ The hallmark clinical and genetic heterogeneity of mitochondrial disease is frequently compounded by the lack of clear genotype–phenotype correlations,^3^ although biochemical assessment of respiratory chain complex activities in skeletal muscle is often helpful in guiding molecular genetic diagnostic testing. For many patients, especially children, the genetic aetiology of their condition remains unknown. Complex IV (also known as cytochrome *c* oxidase (COX)) is the terminal enzyme complex of the mitochondrial respiratory chain, catalysing electron transfer from cytochrome *c* to molecular oxygen, thus contributing to the proton gradient across the inner mitochondrial membrane that drives ATP synthesis.^4^ The human COX enzyme comprises 14 structural subunits, 3 of which are of mitochondrial origin and form the catalytic core.^5,6^ The remaining components are translated on cytosolic ribosomes and imported into mitochondria. The incorporation of all 14 polypeptides to form a mature complex IV is an intricate process orchestrated by over 20 different assembly factors.^5,7^ Recessively inherited defects in several COX assembly proteins result in the failure to assemble a functional holoenzyme and underlie a number of mitochondrial respiratory chain disease presentations characterised by isolated COX deficiency. The clinical manifestation of COX deficiency includes severe myopathy, cardiomyopathy, liver failure and Leigh syndrome, a progressive, subacute, necrotising encephalopathy that is commonly associated with deleterious variants in the *SURF1* gene.^8,9^ SURF1 is an accessory protein related to the yeast *Shy*^10,11^ that facilitates heme *a* insertion into COX1 in the early steps of complex IV biogenesis.^12,13^ Although pathogenic variants in a number of other nuclear-encoded complex IV biogenesis factors have been identified (COA5,^14^ TACO1,^15^ LRPPRC,^16^ COX10,^17^ COX15,^18^ SCO1,^19^ SCO2,^19^ and COX20^20^), the precise mechanism(s) that control COX assembly remain unclear.

Here, we report the application of whole exome sequencing to elucidate the basis of an isolated COX deficiency in a pediatric patient with a severe and fatal neonatal presentation of mitochondrial disease due to a homozygous truncating variant in the *PET100* gene. Previous studies in yeast identified *PET100* gene as a COX biogenesis factor,^21, 22, 23^ and more recently a Lebanese *PET100* founder mutation has been described in 10 individuals presenting with Leigh syndrome.^24^ Fibroblasts and skeletal muscle of our patient showed impaired complex IV activity, associated with a profound defect in COX assembly, and decreased steady-state levels of complex IV proteins. These data provide further evidence that *PET100* is an essential factor involved in the maturation and assembly of complex IV.

## Subjects and methods

### Patient 1

Our patient (ID 73387) is a female child, born by an emergency caesarean section at 34 weeks of gestation to consanguineous, first-cousin British Pakistani parents. Antenatal scans showed that she was small for her gestation, weighing 1.19 kg at birth with a head circumference of 26.7 cm, considerably below the 0.4th centile. Induction of labour had been attempted because of the growth retardation but had failed, leading to the emergency caesarean section. The Apgar scores were 4 at 1 min, 7 at 5 min and 9 at 10 min. She was admitted to the neonatal intensive care unit for continuous positive airway pressure ventilation.

At a few hours of age, she developed a severe lactic acidosis. The initial lactic acid concentration was 22 mmol/l and subsequently increased to 63 mmol/l (normal range, 0.7–2.1 mmol/l). She was treated with intravenous infusions of sodium bicarbonate and Tris-hydroxymethyl aminomethane (THAM), but it was never possible to correct the metabolic acidosis. She also developed hypoglycaemia within hours of birth that was corrected with an intravenous infusion of 15% glucose (7.8 mg/kg/min). The ammonia concentration was normal. Urine organic acid profile showed massive excretion of lactic acid and increased phenolic acids, especially hydroxyphenyllactate. Plasma amino acids showed raised concentrations of alanine and glutamine (1567 and 1369 *μ*mol/l, respectively), consistent with the lactic acidosis; several other amino acids were also mildly increased. There was gross generalised aminoaciduria. Blood acylcarnitine analysis was normal. Echocardiography showed a structurally normal heart and good ventricular function. Cranial ultrasound showed bilateral intraventricular cysts within the frontal horns and anterior portions of the lateral ventricles. The left-sided cysts were larger, up to 15 mm in diameter, whereas the largest cyst on the right was 8 mm in diameter. The choroid plexuses were hyperechoic and irregular, suggesting previous intraventricular haemorrhage. Abdominal ultrasound showed a distended urinary bladder but was otherwise unremarkable. There was severe coagulopathy with an extended prothrombin time of 47.7 s (normal 12.3–16.6 s), a very low plasma albumin of 7 g/l (normal 35–50 g/l), otherwise normal liver function tests but a raised creatine kinase of 2700 U/l (normal <300 U/l).

She was transferred to a tertiary centre because of her worsening metabolic acidosis. She started having seizures at ∼48 h of age. Despite infusions of bicarbonate and THAM, her acidosis continued to worsen. Muscle and skin biopsies were performed and the family agreed to the withdrawal of intensive care treatment. She died aged 55 h. All documented studies were approved and performed under the ethical guidelines issued by each of our Institutions for clinical studies, with written informed consent obtained from the family.

### Cell culture

Fibroblasts from the affected individual and age-matched controls were cultured in Eagle's minimal essential medium (Sigma, Gillingham, UK) supplemented with 10% (v/v) fetal calf serum, 1 × non-essential amino acids, 1 mM sodium pyruvate and 50 *μ*g/ml uridine, humidified at 37 °C and 5% CO_2_.

### Muscle histology and biochemistry

Informed consents with appropriate ethics review committee approvals were obtained. Histological and histochemical analyses were performed on 10 *μ*m transversely orientated serial cryosections of skeletal muscle biopsy samples using standard procedures. The activities of individual respiratory chain complex activities and citrate synthase, a mitochondrial matrix marker, were determined in muscle homogenates and cultured skin fibroblasts as previously described.^25^

### Molecular genetics

Total genomic DNA was obtained using standard methods and the coding region plus intron–exon boundaries of several COX assembly (*SURF1*, *SCO1*, *SCO2*, *COX10*, *COX14*, *COX15*, *COA5*, *LRPPRC*, *TACO1*, *FAM37A*) and structural (*NDUFA4*) genes were amplified using locus-specific primers (sequences available upon request), sequenced using the BigDye v3.1 kit and capillary electrophoresed on the ABI3130xl fluorescent sequencing platform (Life Technologies, Warrington, UK).

Whole exome sequencing was undertaken to investigate the genetic basis of this child's mitochondrial disease presentation as previously described.^26^ A SureSelect Human All Exon 50 Mb V5 Kit (Agilent, Santa Clara, CA, USA) was used for enrichment of coding DNA fragments and sequencing was performed on a HiSeq2000 system (Illumina, San Diego, CA, USA). BWA (version 0.5.8) was used for read alignment to the human reference assembly (hg19) and single-nucleotide variants (SNVs) and small insertions and deletions were detected with SAMtools (version 0.1.7). The average coverage was 128-fold and >97% of the target region was covered at least 20-fold allowing for high-confidence variant calls. Detailed sequencing statistics are provided in Table 1.

### Cell lyses and western blotting

Cultured fibroblasts were harvested and lysed in 50 mM Tris-HCl pH 7.5, 130 mM NaCl, 2 mM MgCl_2,_ 1 mM phenylmethanesulfonyl fluoride (PMSF), 1% Nonidet P-40 (v/v) and 1 × EDTA free protease inhibitor cocktail (Pierce, Rockford, IL, USA). Protein lysates (40 *μ*g) were separated according to size on 12% gels by sodium dodecyl sulphate—polyacrylamide gel electrophoresis (SDS-PAGE) and electrophoretically transferred to a PVDF membrane (Immobilon-P, Millipore Corporation, Darmstadt, Germany). Immunoblotting was performed using primary and HRP-conjugated secondary antibodies.

### Mitochondrial preparation and blue native electrophoresis

Cultured fibroblasts were harvested, resuspended in homogenisation buffer (HB) (0.6 M mannitol, 1 mM ethylene glycol tetraacetic acid, 10 mM Tris-HCl pH 7.4, 1 mM PMSF and 0.1% (v/v) bovine serum albumin (BSA)) and subjected to 3 × 15 passes of homogenisation using a Teflon glass Dounce homogeniser at 4 °C. Standard differential centrifugation (400 *g* for 10 min) was used to remove nuclei and cell debris and mitochondria were finally pelleted at 11 000 *g* for 10 min at 4 °C. Mitochondria were washed in HB without BSA and the final pellet was solubilised by *n*-Dodecyl *β*-D-maltoside (DDM) (Sigma) at 2 mg/mg protein on ice for 20 min. Following centrifugation (100 000 *g* for 15 min at 4 °C) the supernatant was collected and Coomassie Blue G-250 (AMS Biotechnology (Europe) Ltd, Abingdon, UK) was added. Mitochondrial membrane proteins (50 *μ*g) were loaded on a NativePAGE 4–16% BisTris gel (Life Technologies), electrophoretically separated and transferred to a PVDF membrane. The membrane was subsequently immunoblotted with antibodies raised against OXPHOS complexes.

### Immunoblotting

The following primary antibodies were used for immunoblotting: NDUFA9 (Molecular Probes, Eugene, OR, USA, A21344), NDUFB8 (Abcam, Cambridge, UK, ab110242), SDHA (MitoSciences, Eugene, OR, USA, MS204), UQCRC2 (Abcam, ab14745), COX1 (Abcam, ab14705) and COX2 (Molecular Probes, A6404), ATP5A (Abcam, ab14748), ATPB (Abcam, ab14730) and TOM20 (Santa Cruz, Heidelberg, Germany, sc11415). HRP-conjugated anti-mouse or anti-rabbit secondary antibodies were used (P0260 and P0399 respectively; Dako, Glostrup, Denmark). Chemiluminescence ECL Prime Kit (Amersham, Little Chalfont, UK) and ChemiDocMP Imaging System (Bio-Rad, Hemel Hempstead, UK) were used for signal detection and Image lab 4.0.1 (Bio-Rad) software for analysis.

## Results

### Muscle histochemistry and respiratory chain analyses

**Figure 1:**
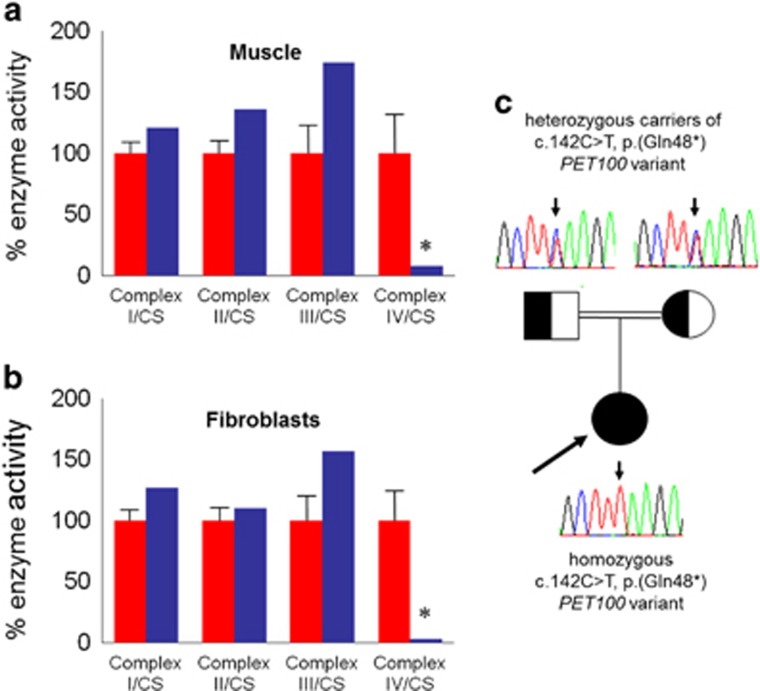
Identification of an isolated mitochondrial respiratory chain complex IV deficiency in muscle and fibroblasts and analysis of *PET100* variant. The assessment of individual respiratory chain enzyme activities in muscle (**a**) and fibroblasts (**b**) identified a severe OXPHOS deficiency affecting complex IV in isolation in the patient (blue bars) compared with controls (red bars); mean enzyme activities shown for muscle controls (*n*=25) and fibroblast controls (*n*=10) are set at 100%. (**c**) Family pedigree showing confirmation of p.(Gln48*) carrier status in clinically unaffected parents, whereas the proband is homozygous for the truncating variant.

Analysis of the patient's muscle biopsy demonstrated a severe and global loss of COX histochemical activity throughout the section (not shown), confirmed by the spectrophotometric assay of respiratory chain activities, that demonstrated a severe and isolated deficiency of complex IV in muscle homogenates (Figure 1a). This observation was confirmed in patient fibroblasts in which complex IV activity was markedly decreased (Figure 1b).

### Molecular genetic studies identify a novel truncating PET100 variant

Sanger sequencing of several COX assembly genes and structural components did not identify causative variants, prompting whole exome sequencing. Prioritisation of candidate disease genes was performed essentially as reported previously,^27^ with our analysis focussing on nonsynonymous variants. Based on the rare disease phenotype we expected disease-causal variants to have a low frequency in the general population, and hence we excluded detected variants present in 3600 control exomes and public databases. Assuming an autosomal recessive mode of inheritance, we searched for genes carrying predicted compound heterozygous or homozygous variants (Table 1). This filter left 38 genes. Two genes, namely *MYH14* (MIM*608568) and *ANKS6* (MIM*615370), have been previously linked to human disease. Variants in both were excluded as likely candidates because of different clinical presentations, reportedly autosomal dominant mode of inheritance in the case of *MYH14*, and the fact that both heterozygous *ANKS6* variants were confirmed to be *in cis* on the same allele. Only one gene, *PET100* (NM_001171155.1), carried two predicted loss-of-function alleles. The patient was homozygous, for a truncating *PET100* variant (c.[142C>T][142C>T], p.[(Gln48*)][(Gln48*)] ClinVar Reference ID: mdi-3317) that resides in the fourth coding exon and predicts a truncated protein in which the last 26 amino acids are lost (33% of the full-length protein). Concordant with a disease-causal role of the homozygous (c.142C>T, p.(Gln48*)) variant, confirmatory Sanger sequencing revealed that both healthy parents were heterozygous carriers (Figure 1c).

### Mutation of PET100 leads to impaired complex IV assembly

Further characterisation of the nature of the biochemical defect associated with the PET100 variant was performed in patient fibroblasts. The steady-state levels of individual OXPHOS complex subunits and the subsequent assembly into mitochondrial respiratory chain complexes were analyzed by Blue-native PAGE (BN-PAGE) and SDS-PAGE respectively.

**Figure 2:**
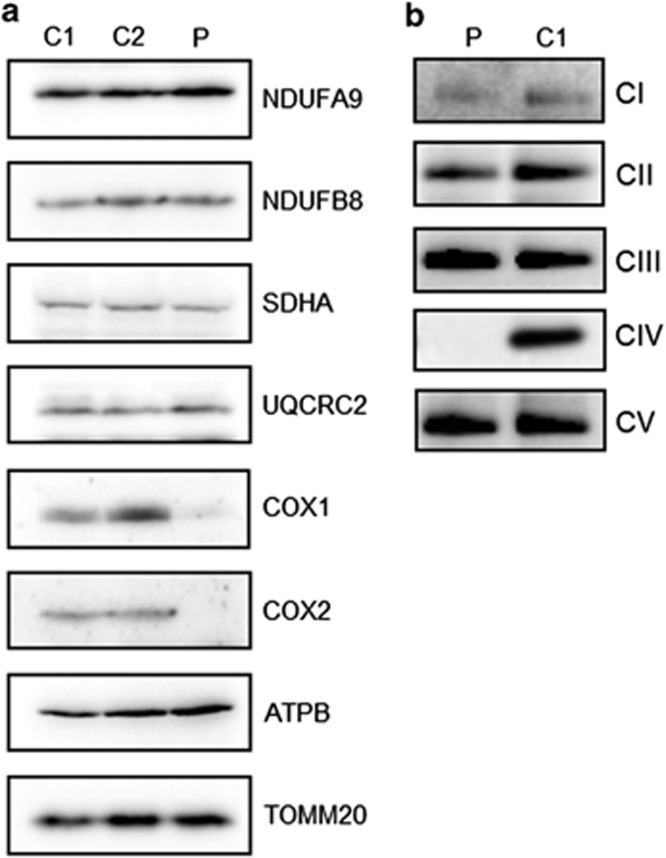
Steady-state levels of OXPHOS components and complexes. (**a**) Cell lysate from control (C1 and C2) and patient (P) fibroblasts (40 *μ*g) were analysed by SDS-PAGE (12%) and immunoblotting. Subunit-specific antibodies were used against CI (NDUFA9, NDUFB8), CII (SDHA), CIII (UQCRC2), CIV (COX1, COX2) and CV (ATPB). The outer mitochondrial membrane marker, TOM20, was used as a loading control. (**b**) Mitochondrial proteins (50 *μ*g) isolated from patient (P) and control (C1) fibroblasts were analysed by one-dimensional BN-PAGE (4 to 16% gradient) using subunit-specific antibodies as indicated (CI (NDUFA9), CII (SDHA), CIII (UQCRC2), CIV (COX1) and CV (ATP5A)) to assess the assembly of individual OXPHOS complexes. Complex II (SDHA) was used as a loading control.

The analysis of the steady-state levels of OXPHOS complex components confirmed a marked decrease in COXI and COXII in patient compared with control fibroblasts (Figure 2a). No significant changes were detected in any of the steady-state levels of all other analyzed complexes (I, II, III and V), although in agreement with the respiratory chain enzyme results in muscle, there was a suggestion that complex III levels were increased ~1.6-fold based on densitometric analysis (Figure 2a). A similar observation has been made previously in patients harbouring pathogenic variants in another COX assembly gene, *SURF1*.^28^ TOM20 was used as a mitochondrial loading marker and confirmed equal loading of control and patient mitochondrial protein.

Consistent with this reduction in steady-state levels of CIV proteins and the biochemical measurements of the respiratory chain complex activities (Figure 1b), BN-PAGE analysis revealed significantly decreased amounts of fully assembled complex IV in patient cells compared with age-matched controls (Figure 2b). This loss of OXPHOS complex was specific as the assembly profile of complexes I, II, III and V were normal.

These data demonstrate that the consequence of the homozygous p.(Gln48*) PET100 variant is a specific and severe loss of COX subunits and fully assembled complex IV.

## Discussion

Although recognised as one of the most common energy metabolism disorders, isolated COX deficiency has a diverse genetic aetiology that reflects the complex nature of biogenesis and assembly of mtDNA- and nuclear-encoded components into mature holoenzyme; a process facilitated by numerous chaperone proteins. Pathogenic variants in a number of the assembly factors necessary for the formation of a functional COX enzyme have been reported.^9,14, 15, 16, 17, 18, 19, 20^ Recently, a founder mutation in a highly conserved COX assembly factor *PET100* has been identified in 10 Lebanese individuals with isolated COX deficiency who present with Leigh syndrome and seizures.^24^

Here we report that a new truncating *PET100* variant causes fatal infantile lactic acidosis and isolated COX deficiency in a child born to consanguineous British Pakistani parents. The pathogenic nonsense (c.142C>T, p.(Gln48*)) variant in the *PET100* gene was identified by whole exome sequencing, leading to impaired complex IV enzyme activity and abnormal COX assembly. Our results are consistent with previously published data suggesting that *PET100* is a conserved biogenesis factor involved in the maturation of complex IV in both humans^24^ and yeast.^21, 22, 23^ The yeast homologue of *PET100* is not necessary for the localisation of COX polypeptides to the inner mitochondrial membrane,^21^ but it has a major role in the later assembly processes where it facilitates the assembly of COX intermediates.^23^ In contrast, human *PET100* appears to be required earlier in the process for the assembly of mitochondrial-encoded COX subunits.^24^ Our results demonstrate the importance of *PET100* in OXPHOS function and support previous studies;^21, 22, 23, 24^ however, it requires further investigation to fully understand the exact role of this enzyme in the maturation of the COX holoenzyme.

The complex IV assembly profile observed in our patient with this truncating *PET100* variant is similar to the reported Lebanese (c.3G>C, p.?) *PET100* variant that eliminates the initiation codon potentially resulting in a nonfunctional protein.^24^ However, our study has identified some key differences in the biochemical and clinical disease presentations between the two variants. The residual complex IV enzyme activities were lower in our patient's fibroblasts and skeletal muscle compared with the residual COX activities demonstrated in tissues from the Lebanese patients. The COX defect in the patients carrying the Lebanese (c.3G>C, p.?) variant was associated with Leigh syndrome, seizures, developmental delay and elevated blood lactate levels, although these were variable (ranging from normal to 11 mmol/l).^24^ These symptoms were apparent a few months after birth. In contrast, the onset of the disease in our patient was before birth and her lactate levels were extremely high (63 mmol/l at its peak). Further differences in our patient's clinical presentation were marked hypoglycaemia, severely impaired liver function and raised creatine kinase reflecting profound disruption of metabolic energy homeostasis. These observations in our patient suggest that impairment of *PET100* can lead to severe complications, including prenatal onset and neonatal death, not observed in the other reported *PET100* variant. Interestingly, Lebanese individuals harbouring the *PET100* truncating variant differ from patients with mutations in *SURF1*, a different COX assembly factor, in that seizures appear to have an earlier age of onset.^24,29^ Consistent with this, our microcephalic patient showed abnormalities on neuroimaging and suffered seizures from 48 h of age that are likely to reflect severe problems with *in utero* brain development. The truncating nature of the *PET100* variant may cause the protein to be subject to nonsense-mediated mRNA decay or may otherwise exert a dominant negative effect that in turn determines the severity and early appearance of clinical disease. Importantly, although a *PET100* variant has only been identified in patients originating from Lebanon to date, our patient shows that mutations within this gene occur outside of this particular ethnic group.

Whole exome sequencing is a rapid and effective approach to elucidate the molecular bases of mitochondrial respiratory chain disorders including isolated COX deficiency.^30,31^ Our findings confirm *PET100* as an important candidate disease gene in patients with isolated COX deficiency. Recent advances in next-generation sequencing enable the rapid and accurate diagnosis of singleton mitochondrial disease patients within small families, thus facilitating appropriate counselling and the offer of preventive strategies, such as prenatal diagnosis and preimplantation genetic profiling.

## Figures and Tables

**Table 1:** Variants identified at different filtering levels in individual no. 73387

*Variants filtering*	
Synonymous variants	11 836
Nonsynonymous variants (NSVs)	12 576
NSVs absent from 3600 control exomes and public databases	313
Genes carrying ≥2 NSVs	38
Genes carrying ≥2 loss-of-function alleles	1 (***PET100***)

NSVs indicate missense, nonsense, stop/loss, splice site disruption, insertions and deletions. The bold entry indicates the affected gene (*PET100*).
